# Estimating trends in working life expectancy based on health insurance data from Germany – Challenges and advantages

**DOI:** 10.1016/j.ssmph.2022.101215

**Published:** 2022-08-28

**Authors:** J. Tetzlaff, M. Luy, J. Epping, S. Geyer, J. Beller, J.T. Stahmeyer, S. Sperlich, F. Tetzlaff

**Affiliations:** a Medical Sociology Unit, Hannover Medical School, Hannover, Germany; b Vienna Institute of Demography, Austrian Academy of Sciences, Vienna, Austria; c Wittgenstein Centre for Demography and Global Human Capital (IIASA, OeAW, University of Vienna), Vienna, Austria; d AOK Niedersachsen- Statutory Health Insurance of Lower Saxony, Hannover, Germany; e Division of Social Determinants of Health, Robert Koch-Institute, Berlin, Germany

**Keywords:** Working life expectancy, Time trend, Educational inequality, Health insurance data, Germany

## Abstract

Against the backdrop of population aging and growing strain on pension systems, monitoring the development of Working Life Expectancy (WLE) is vital to assess whether the policies taken are effective. This is the first study investigating time trends and educational inequalities in WLE based on German health insurance data. The analyses are based on the data of the AOK Lower Saxony (N = 3,347,912) covering three time periods (2006-08, 2011-13, and 2016-18). WLE is defined as years spent in the labor force (i.e. in employment and unemployment) and was calculated for each age between 18 and 69 years for the three periods to depict changes over time using multistate life table analysis. Educational inequalities in 2011-13 are reported for two educational levels (8–11 years and 12–13 years of schooling). WLE increased in both sexes with increases being stronger among women. This holds irrespective of whether WLE at age 18 (35.8–38.3 years in men, 27.5–34.0 years in women) or the older working-age (e.g. at age 50 10.2–11.7 years in men, 7.8–10.5 years in men) is considered. Among women at all ages and men from their mid-20s onwards, WLE was higher among higher-educated individuals. Inequalities were most pronounced among women (e.g. Δ3.1 years in women, Δ1.3 years in men at age 50). The study supports previous research indicating that measures to extend working life are effective, but that noticeable inequalities in WLE exist. Health insurance data represent a valuable source for such research that has so far remained untapped. The data provide a suitable basis to investigate trends and inequalities in WLE. Future research should build on the strengths of the data by broadening the research towards a more comprehensive analysis of the development of WLE from a health perspective.

## Highlights

• Working Life Expectancy has increased substantially since the mid-2000s.
• The gender gap narrowed over time, but men still spend considerably more years in the labor force than women.
• Educational inequalities in WLE exist in both sexes, but are larger in women.
• Measures to increase labor force participation should focus especially on lower-educated women, as WLE is low in this group.
• Prevention should be strengthened for vulnerable groups to reduce health inequalities and early labor market exits.

## 1 Introduction

Increasing life expectancy and population ageing are putting growing strain on public pension systems in high-income countries. Therefore, in recent decades measures have been taken in many countries to ease this burden by prolonging working lives and increasing the ratio of the working to the non-working population (Anderson et al., 2019; Hytti & Valaste, 2009). Among them, Germany is one of the demographically oldest countries with a median age of 44.1 years for men and 47.6 years for women in 2019 (Bundesinstitut für Bevölkerungsforschung, 2022). Policies responded by gradually raising the regular retirement age from 65 to 67, which will be reached in 2031. Early retirement leads to lower pension payments, but is frequently used in Germany. Therefore, the actual retirement age due to old age was 64 years on average in 2021 (Deutsche Rentenversicherung, 2022). Against this background, monitoring the development of the length of working life over time is becoming increasingly important, as this can provide information on whether the political measures to extend working life are effective (Loichinger & Weber, 2016). Furthermore, the question arises as to how large the existing social inequalities in working life expectancy are and how they have developed over time.

Studies investigating trends in the length of working life usually draw conclusions from analyzing the development of working life expectancy (WLE). This indicator depicts the number of (remaining) years expected to be spend in economically active states (Hoem, 1977). Previous research differs with respect to the definition of WLE (years in paid employment vs. years in the labour force) and to the methods applied (i.e. multistate life tables vs. Sullivan method (Sullivan, 1971)). Therefore, direct comparisons between studies are often hampered especially if exact values of WLE should be compared. However, irrespective of these limitations previous European studies examining the development of WLE of the older working-age population consistently reported distinct increases in recent decades for both sexes, with increases being most pronounced in women (Leinonen et al., 2018; Loichinger & Weber, 2016; Nurminen, 2012; van der Noordt et al., 2019; Weber & Loichinger, 2020). This holds irrespective of whether WLE is defined based on active labor (e.g. Robroek et al., 2020)) or on labor force participation (i.e. years in employment and unemployment (e.g. (Eurostat, 2022a; Loichinger & Weber, 2016)), indicating that time trends are similar and general trends can be compared. This development was mainly driven by increased labor force participation at age 50 and older (Loichinger & Weber, 2016). Previous research has shown that WLE varies considerably by education (Loichinger & Weber, 2016; Robroek et al., 2020; van der Noordt et al., 2019; Weber & Loichinger, 2020) and occupational group (Kadefors et al., 2019; Leinonen et al., 2018; Schram et al., 2021), with individuals with lower socioeconomic status having lower WLE. Since current political measures primarily aim at increasing the labor force participation in the elderly, most studies on WLE focus on the older population (mostly age 50+). However, against the background of prolonged training periods and increasing female employment across the entire range of working-age, it is also important to investigate changes in WLE at the beginning of the working biography as well.

Previous research investigating time trends or social inequalities in WLE has been primarily based on survey data (e.g. Dudel et al., 2021; Loichinger & Weber, 2016; Nurminen, 2012; Weber & Loichinger, 2020)). For Germany, studies on trends in WLE reported substantial increases at age 15 (Eurostat, 2022) and 18 (Heller et al., 2022) as well as at age 50 and above (Heller et al., 2022; Loichinger & Weber, 2016; Weber & Loichinger, 2020) during the last two decades. So far, however, evidence on social inequalities in WLE in Germany is still very limited. This is due to the lack of official life tables by socio-economic characteristics in Germany, which are often used to calculate WLE based on survey data. To the best of our knowledge there is so far only one study which investigated time trends in paid full-time equivalent WLE at age 55 to 64 in Germany, ignoring the effect of mortality on WLE (Dudel et al., 2021). This study found that educational inequalities in WLE are substantial and that disparities have increased, at least in East Germany (Dudel et al., 2021).

This raises the question of whether other data sources can also can be used to study trends and inequalities in WLE. For Germany, the use of statutory health insurance data is conceivable since information on employment status and mortality are very well recorded and socioeconomic information such as education, occupation and income is also available. The aim of this paper is to describe the possibilities of using health insurance data to estimate the WLE and to analyze time trends and social inequalities in WLE based on this kind of data. In this study, WLE is defined as years spent in the labor force rather than in paid employment only in order to depict trends in years potentially spent in active labor. Given the ageing population, labor shortages are expected to worsen in the future. WLE defined as the years in the labor force provide insight into the potential years in active employment available as the labor market adapts to older workers.

Based on this, our study contributes to the current research by addressing the following questions:

• How did WLE develop over time?
• Did the time trends in WLE differ by gender?
• How large is the gap in working life expectancy between educational groups?

In particular, it will be discussed how the general level of WLE as estimated from German health insurance data can be compared to the WLE calculated from German survey data. Furthermore, it will be considered how the time trends in WLE vary over time between the different data sources. Due to the limited number of studies on social inequalities in WLE in Germany, an additional focus is on the calculation of WLE by education. Here, the advantages and disadvantages of using health insurance data for the calculation of social inequalities in WLE are addressed in detail.

## 2 Methods

### 2.1 Data

The analyses are based on the data of the statutory health insurance provider (AOK Lower Saxony, AOKN) of three time periods (2006–2008, 2011–2013, and 2016–2018). Statutory health insurance is an essential element of the social security system in Germany. Due to legal regulations restricting the access to private health insurance, about 90% of the German population are insured with a statutory health insurance provider (electronic report).

The dataset contains records of approximately two million insured individuals aged 18 years and above per year (in total N = 3,347,912 in all three periods) and cover about a third of the population of Lower Saxony (AOK Niedersachsen, 2019), Germany. The data were collected for accounting purposes and contain longitudinal information on insurance histories, diagnoses, medical procedures and mortality, as well as on employment and unemployment periods and other socioeconomic characteristics, e.g. income and educational level. With respect to sex and age, distributions within the AOKN population is comparable to the total population of Lower Saxony and Germany (Jaunzeme et al., 2013) but differ in terms of occupational groups and educational level with higher levels being underrepresented (Epping et al., 2021). Table 1 displays some basic characteristics of the study population.

**Table 1:** Descriptive statistics of the study population: the number of individuals, events, and person-years by sex.

		Men			Women		
		Individuals	Person-years	Events	Individuals	Person-years	Events
**Period**	2006–2008	833,608 (29%)	2,113,997 (29%)	.	823,031 (30%)	2,125,703 (30%)	.
	2011–2013	940,068 (33%)	2,420,131 (33%)	.	879,567 (32%)	2,321,750 (33%)	.
	2016–2018	1,109,059 (38%)	2,737,204 (38%)	.	1,019,454 (37%)	2,598,409 (37%)	.
**Education**^a^	low	560,780 (60%)	1,501,377 (62%)	.	499,631 (57%)	1,359,509 (59%)	.
	high	93,000 (10%)	235,775 (10%)	.	110,695 (13%)	283,417 (12%)	.
	no certificate or unknown	286,288 (30%)	682,985 (28%)	.	269,241 (31%)	678,825 (29%)	.
**Transition**	non-labor force to labor force	.	.	360,857	.	.	395,019
	labor force to non-labor force	.	.	376,046	.	.	361,743
	non- labor force to death	.	.	28,166	.	.	16,970
	labor force to death	.	.	15,823	.	.	4993

Note: Percentages do not always add up to 100% due to rounding; Data source: AOK Lower Saxony health insurance data.

a Values refer to the middle period 2011–2013 only (for more information, see section “educational information”).

### 2.2 Labor force definition

In previous research, the labor force concept of the International Labor Force Organization (ILO) (Benes, 2018; European Commission, 2016) has been applied to calculate WLE (e.g. (Loichinger & Weber, 2016; Weber & Loichinger, 2020)). According to this definition, all employed individuals are counted as part of the labor force as well as individuals who are employed but temporarily not working due to illness, parental leave, further training, etc. In addition, all unemployed individuals are assigned to the labor force if they are actively seeking employment and are quickly available to start a new job (Benes, 2018; European Commission, 2016). Accordingly, the labor force represents not only the employed, but also the population which would potentially be available to the labor market. In line with the ILO-definition, episodes of paid employment (including self-employment) and unemployment (i.e. receiving unemployment benefits) were defined as episodes contributing to the time an individual spent in the labor force. In contrast, episodes of retirement (e.g. due to old age or disability) or of family insurance (i.e. episodes without income during which spouses and children below age 23 can be co-insured free of additional fees with a working married partner or a parent) were assigned to the periods spend in the non-labor force. Further details on the assignment of the different episodes can be found in the online supplement (Online Resource 1, additional remarks 2). Whenever there were overlapping episodes of employment and unemployment, the person was defined to be economically active and thus the episode contributed to the lifespan spent in the labor force.

### 2.3 Educational information

Information on educational attainment is available for insured individuals who were ever employed within the observation period (in this case 2005 to 2018). For these analyses we used the highest school-leaving qualification, since school education is usually completed at age 18 to 19 and is therefore suitable for the analysis of WLE at age 18 and above. If education was missing for the current episode (e.g. due to unemployment or missing data), the educational information from the previous or the following episodes was assigned. Three educational groups can be distinguished: (1) graduation after 8–11 years of schooling (Volksschule, Hauptschule, Realschule or equivalent, low), (2) after 12–13 years of schooling (Abitur, Fachabitur or equivalent, high), and (3) no school-leaving qualification or unknown qualification (Table 1). Inequalities were analyzed by comparing WLE of the low- and the high-educated group.

Since education is only coded for insured individuals who have ever been employed during the observation period, it is especially often lacking for individuals with long-term family insurance or unemployment episodes. Therefore, the total percentage of missing information on education is considerably higher in the non-labor force (57%) than in the employed individuals (27%). In order to reduce the share of missing values among the non-employed population, we transferred the educational information of the working spouse or parent to the non-working spouse or child aged 18 years and above with missing information. These strategy was based on the assumption of educational homogamy within married parters (Blackwell & Lichter, 2004; Muschik et al., 2015; Safieddine et al., 2020) and that the educational qualification and that the educational qualifications of parents and children are often comparable (Björklund & Salvanes, 2011; Black et al., 2005; Dickson et al., 2016; Sirin, 2005). Following these strategies, the proportion of missing values among the non-labor force decreased by 16 percentage points. Individuals who still had missing information on education were excluded from the analyses of educational inequalities in WLE. However, due to the short time remaining in gainful employment at older working age, the educational information is often missing at high working age in the first period while it was often derived from earlier years for the elderly in last period. In a similar way, this applies to the very young working age in the last period. Due to this uneven distribution of missing values across age between periods, the analyses on educational inequalities in WLE are limited to the middle period 2011–2013 in which the proportion of missing values on education is lowest (Online Resource 1, Table A5).

### 2.4 Statistical analyses

First, the proportions of labor force participation by period and single-year age group were calculated to depict the general trend in the labor force participation over time and which age groups contributed strongest to this development. To illustrate educational differences across age the labor force proportions by education were calculated as well.

Working life expectancy (WLE) was calculated based on multistate life table analysis. These life tables include two transit states (labor force, non-labor force) allowing for repeated transitions between them and one competing absorbing state (death). Between the three states, four transitions are possible: 1) non-labor force to labor force, 2) labor force to non-labor force, 3) non-labor force to death, and 4) labor force to death (Table 1). In order to describe the temporal development of the transition risks between these three states, cox-proportional hazard models were estimated for each of the three periods. These models contain period as a categorical covariate, are stratified by sex and controlled for age (in single-year age groups). Moreover, age-specific transition rates were calculated. These rates were used as input for the multistate life table analysis which is based on matrix multiplication as described by Palloni (2001, pp. 256–272). WLE was calculated for the three periods 2006–2008, 2011–2013, and 2016–2018. The calculation of WLE is based on partial life expectancy for ages 18–69, since the proportion of individuals being in labor above that age is very low (<1%) in both sexes. The analyses were stratified by sex. WLE is reported at age 18, 50 and 60 to capture differences in trends over time across age. Finally, the differences in WLE are analyzed according to the highest school-leaving qualification for the period 2011–2013.

Data management and the regression models were performed using Stata MP 14.2 (Stata Corp, 2015), the WLE were calculated using R 3.5.1 (RCore Team, 2015). Confidence intervals were obtained from 1000 bootstrap samples (with replacement).

## 3 Results

### 3.1 Time trends and educational differences in the labor force participation

**Fig. 1:**
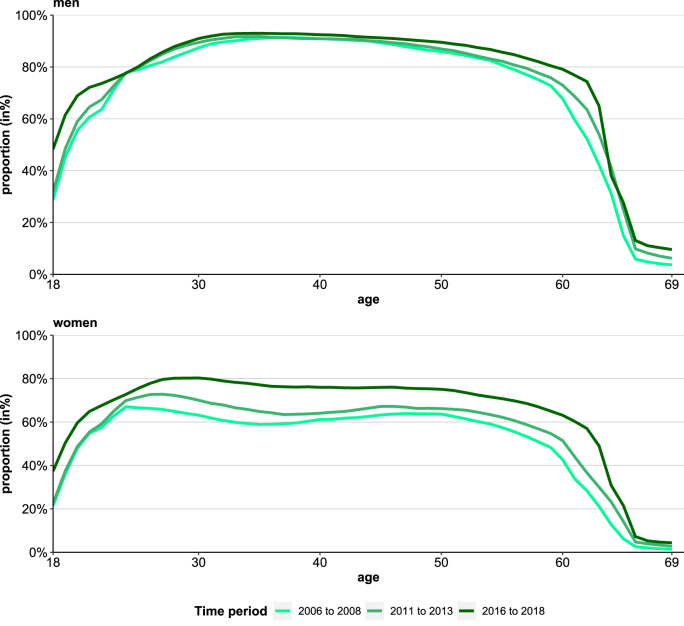
Labor force proportions across age by sex and period Data source: AOK Lower Saxony health insurance data.

**Fig. 2:**
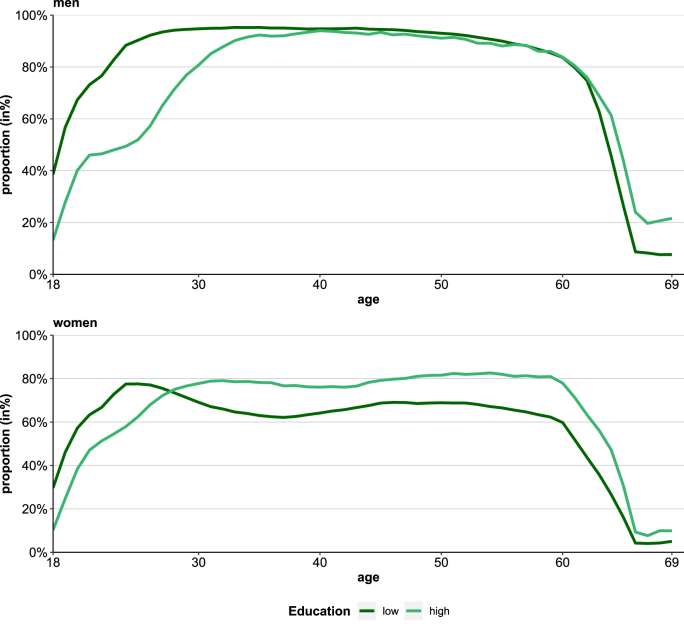
Labor force proportion across age by sex and educational group Data source: AOK Lower Saxony health insurance data.

Between 2006-2008 and 2016-2018e, the total labor force proportion of the insurance population rose in men (72%–79%) and women (50%–66%). Among men, this increase was strongest among the older working-age population above the age of 50. While for women the overall level of the labor force proportion was lower, the extent of increases were much higher than in men across the full age range. Similar to men, the labor force participation increased strongest at higher working ages (Fig. 1). The labor force proportion is higher among women with higher education in all ages (in total 69% vs. 62%). Among younger men, by contrast, the proportion is higher in individuals with lower education (40% vs. 17% at age 18), reflecting their earlier labor market entries (Fig. 2).

### 3.2 Time trends in transition risks

For both sexes, the rates of entering the labor force increased over time. This increase was stronger in women (HR 1.57, Ref. = period 1) than in men (HR 1.38, Ref. = period 1). In contrast, the risk of the reverse transition from the labor force to the non-labor force increased slightly in men (HR 1.05), while it decreased slightly in women (HR 0.94). Among the non-labor force, the competing risk of death remained quite constant in men but increased in women while death risks decreased for economically active men and women (Table 2).

**Table 2:** Time trends in transition risks (Hazard Ratios) by sex.

		Men		Women	
		HR		HR	
**non-labor force to labor force**	2006–2008	1		1	
	2011–2013	1.14	(1.13–1.15)	1.21	(1.20–1.22)
	2016–2018	1.38	(1.36–1.39)	1.57	(1.56–1.58)
**labor force to non-labor force**	2006–2008	1		1	
	2011–2013	1.03	(1.02–1.04)	0.99	(0.98–0.99)
	2016–2018	1.05	(1.04–1.06)	0.94	(0.93–0.94)
**non-labor force to death**	2006–2008	1		1	
	2011–2013	1.05	(1.02–1.08)	1.11	(1.07–1.16)
	2016–2018	1.04	(1.01–1.07)	1.19	(1.15–1.23)
**labor force to death**	2006–2008	1		1	
	2011–2013	0.88	(0.85–0.92)	0.92	(0.85–0.98)
	2016–2018	0.85	(0.82–0.88)	0.86	(0.81–0.92)

Note: all models are controlled for age in single-year age groups, 95%-confidence intervals are given in parentheses, HR Hazard Ratio; Data source: AOK Lower Saxony health insurance data.

### 3.3 Time trends and educational differences in working life expectancy

**Fig. 3:**
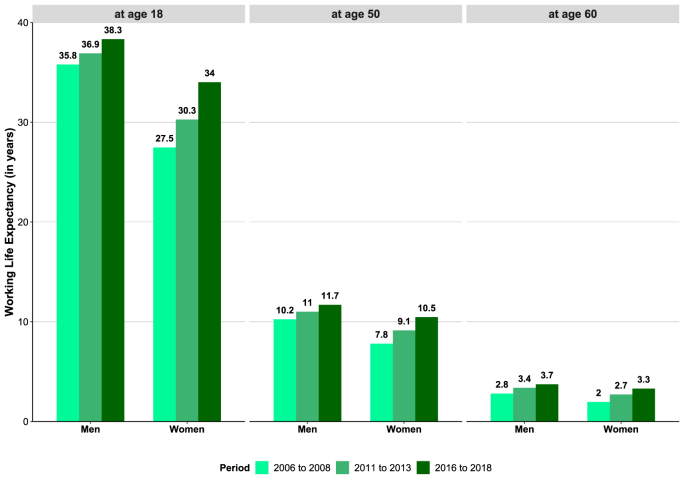
Working Life Expectancy at age 18, 50 and 60 by sex and period Note: Increases in WLE over time are statistically significant; 95%-CIs are displayed in Online Resource 1, Table A1 Data source: AOK Lower Saxony health insurance data.

In the insurance population, the number of economically active life years at age 18 increased from 35.8 years to 38.3 years for men and from 27.5 to 34.0 years for women. WLE also increased substantially at ages 50 (10.3–11.7 years in men, 7.8–10.5 years in women) and 60 (2.8–3.7 years in men, 2.0–3.3 years in women). The stronger increase in WLE among women is also evident at higher working age (age 50: +2.7 years compared to +1.5 years in men) (Fig. 3). All increases in WLE over time are statistically significant (Online Resource 1, Table A1). Analyzing trends across the full range of working age, it becomes apparent that WLE increased substantially at every age up to the highest working ages (Online Resource 1, Fig. A2).

**Fig. 4:**
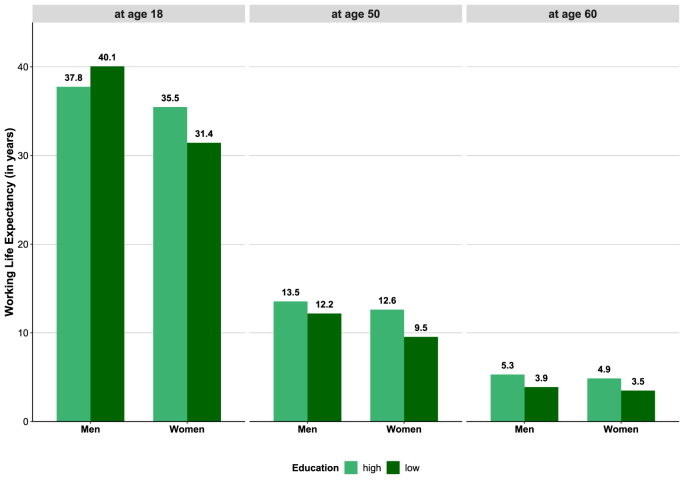
Working Life Expectancy at age 18, 50, and 60 in 2011–2013 by sex and educational group Note: Differences in WLE between educational groups are statistically significant, 95%-CIs are displayed in Online Resource 1, Table A4. Individuals with missing information on education were excluded Data source: AOK Lower Saxony health insurance data.

Furthermore, WLE differs by educational group. For men, these inequalities vary with respect to age. While WLE at age 18 is higher among low-educated than among higher-educated men (Δ 2.5 years), it is lower at older working age (Δ 1.3 years at age 50 and Δ1.1 years at age 60). However, when considering the entire age range from 18 to 69 years, it becomes apparent that men with higher education consistently have a higher WLE from their mid-20s onwards than men with lower educational attainment of the same age (Fig. A3, Online Resource 1). This is because higher educated men stay longer in the labor market than men with low education (Fig. 2). Stronger inequalities were found among women, pointing in the same direction over the entire age range, with WLE being higher among higher-educated women than among women with low education (Δ +4.1 years at age 18, Δ +3.1 years at age 50, Δ +1.6 years at age 60) (Fig. 4, Fig. A3 Online Resource 1).

## 4 Discussion

This study is one of the few studies analyzing time trends and educational differences in WLE in Germany. While previous research has been based on survey data, our study is the first one to use health insurance data. Due to the availability of detailed data on diagnoses and other medical information, health insurance data are a valuable data source to enhance research on the development of the length of working lives from a health perspective.

### 4.1 Main findings

The study shows clear increases in WLE over time for both sexes. These increases were observed in both younger and older working ages and are driven by increasing rates of labor market entries and growing labor force participation, especially in the elderly. Overall, the findings indicate stronger increases in labor force participation and WLE across the entire age range among women than among men. This finding is in accordance with the higher transition rates to labor force and the lower transition rates to non-labor force among women.

Furthermore, the length of working lives differs by educational level. Younger men with low educational level had higher WLE than men with high educational attainment. This is due to earlier labor market entries among men with low education compared to younger men with higher education. From their mid-twenties onwards, however, WLE is higher among men with higher education. For women with higher education, WLE is higher regardless of the age group considered. While clear inequalities in WLE emerged in both sexes they were more pronounced among women reflecting that women with higher education enter the labor market more frequently and stay economically active up to higher ages than lower-educated women. In addition, the higher level of labor force participation indicates that men tend to be economically active more often irrespective of education.

### 4.2 Discussion in the context of previous research

Previous studies analyzing time trends in WLE in Germany focused mostly on the higher working-age (Loichinger & Weber, 2016; Weber & Loichinger, 2020) and reported mainly partial WLE for specific age ranges (Dudel et al., 2021; Weber & Loichinger, 2020). In addition, there are differences either in the definition of the working population (Dudel et al., 2021) or in the time period studied. Therefore, findings cannot be compared directly across the entire period (Loichinger & Weber, 2016). Nevertheless, comparisons with previous research can provide some insight into whether general trends and educational inequalities in the insurance population differ from the findings reported for the total German population obtained from survey data. Comparing our results with the WLE at age 50 based on the ILO labor force concept, the overall level of WLE tends to be lower than the survey-based results (Heller et al., 2022; Loichinger & Weber, 2016). Similarly, the level of WLE at young working-age is lower in our study than those reported by Eurostat (Eurostat, 2022a) and another study based on the German Socio-economic Panel (Heller et al., 2022). This is in accordance with our expectation and may be explained by the differences in the statistical methods used (Sullivan versus multistate life approaches) and because individuals with low SES (and therefore with low WLE) are overrepresented compared to the general German population. However, although the general level of WLE reported in this paper is lower, comparing the results with previous research shows that the data reflect time trends in WLE in the general German population well. This holds for time trends at younger (Eurostat, 2022a; Heller et al., 2022) and older working age (Dudel et al., 2021; Heller et al., 2022; Loichinger & Weber, 2016; Weber & Loichinger, 2020). In line with previous research, our study shows that WLE increased much stronger among women than among men (Eurostat, 2022a; Heller et al., 2022; Loichinger & Weber, 2016; Weber & Loichinger, 2020). The stronger increase in women led to a narrowing gender gap in WLE over time. However, as in earlier studies, the gap in WLE between men and women persisted and men still work considerably more years than women (Dudel et al., 2021; Eurostat, 2022a; Heller et al., 2022; Loichinger & Weber, 2016; Weber & Loichinger, 2020).

To the best of our knowledge, there is only one previous study investigating social differences in WLE in Germany (Dudel et al., 2021). This study also found substantial disparities in WLE between educational groups. This is consistent with international research, which also reported large inequalities, especially at higher working ages (e.g. (Dudel & Myrskylä, 2017; Leinonen et al., 2018; Loichinger & Weber, 2016; Robroek et al., 2020; Schram et al., 2021; Weber & Loichinger, 2020)). While this previous study reported high educational inequalities at age 55–64 in both sexes (Dudel et al., 2021), our findings indicate much higher inequalities in WLE in women than in men. This difference between men and women in inequality levels between the studies may be explained by methodological reasons, especially due to the differing definitions of working life (e.g. by using employment periods only instead of combining periods of employment and unemployment, ignoring the effect of mortality on WLE, or applying an adjustment of total WLE for working hours to measure full-time equivalent WLE). Since the continuity of employment biographies differs greatly between the SES groups, higher inequalities are to be expected if only the periods of active employment are included. Comparing our results with a recent study indicates that inequalities are significantly greater in terms of years in active employment than in terms of time spent in the labor force, especially for men (Dudel et al., 2021). While studies on WLE based on employment periods alone add to the current knowledge on trend and inequalities in life years spent in active labor (Dudel et al., 2021), the results are influenced by the current economic conditions and the “potential” WLE cannot be captured. Therefore, previous studies also differ in terms of content-related aspects. Our study complements to the current research by reporting educational differences in the length of life spent in the labor force.

Since most research on social inequalities is focused on WLE among the elderly, the evidence is more limited when it comes to social inequalities in WLE at very young working ages (e.g. (Robroek et al., 2020)). The focus on paid employment in previous research may explain why higher WLE at younger working ages was found in both sexes in the group of higher educated, while our results suggest that WLE at age 18 is higher among men with low educational attainment. This may be due that lower-educated men are more likely to enter the labor market through unemployment than men with higher education, which also contribute to WLE following the labor force concept. For women, our results are in line with previous research indicating longer working lives for women with higher education irrespective of the age group considered (Robroek et al., 2020).

### 4.3 Strengths and limitations

#### 4.3.1 Labor force concept

Previous studies define WLE either as the number of years expected to be in the labor force or as years spent in paid work (Dudel et al., 2021; Leinonen et al., 2018; Loichinger & Weber, 2016; Parker et al., 2020a, 2020b; Robroek et al., 2020; Schram et al., 2021; Weber & Loichinger, 2020). While both approaches clearly contribute to the current state of research, the use of the labor force definition allows to describe potential years in employment. This study defines WLE as the number of years spent in the labor force, i.e. periods of employment and unemployment equally contribute to WLE. This definition is particularly suitable to address the question of whether working life has increased independently of the changing economic framework. . Other studies, however, are more interested in the years spent in paid employment, as increases in active employment directly contribute to economic output and ease the burden on pension funds. Accordingly, both definitions contribute to the current state of research and it depends on the research question which definition is to be preferred.

Another advantage of analyzing trends in WLE based on labor force participation is that this approach is less sensitive to short-term period effects which may lead to sudden but temporary drops in WLE, e.g. due to temporary sharp increases in unemployment rates due economic crises. Similar to many previous studies, our analyses are based on period life tables which are prone to such period effects. By explicitly including unemployment episodes, the impact of such effects can be reduced.

#### 4.3.2 Data characteristics relevant to calculate WLE

The calculation of WLE is based on detailed data on labor force and educational attainment, which allowed us to analyze WLE based on different transition rates relevant to the length of working lives. Furthermore, information on mortality are provided in the same dataset. Therefore WLE could be analyzed for the general insurance population without excluding those who died within the study period and is therefore not conditional on surviving to a respective age. This is an advantage especially when WLE is reported for older ages, when deaths occur more frequently. Without taking mortality into account, WLE at general population level would be overestimated.

In this study, labor force is measured following the ILO-definition (Benes, 2018; European Commission, 2016) as far as possible. However, this study differs in two aspects from this definition. First, while the ILO-definition is based on the employment status of a specific reference week received from surveys, labor force participation in our study is measured based on secondary data. This means that the information on different (non-)employment episodes is recorded across the full time period in which the individual is insured with the health insurance provider and labor force histories within these periods can be followed straightforward. The continuity of the labor force information in the data is a strength of the study because it allows for a time-related assignment of employment status, which enabled us to calculate WLE based on multistate life tables. Multistate analyses are considered preferable to the Sullivan method (Sullivan, 1971) frequently used for cross-sectional data, because life expectancies can be estimated more precisely, especially when transition rates fluctuate over time (Jagger et al., 2014; Mathers, 1991). Second, the health insurance data do not provide information on whether the unemployed insured individuals actually intend to achieve gainful employment. In contrast to the ILO-definition, we could therefore not distinguish between persons who are actively seeking for employment and who are ready to take up an offered job within the next two weeks and those who are not. Therefore, WLE might be somewhat overestimated compared to the results that would have been obtained if the calculation of the WLE was based on the original ILO labor force definition. However, since the labor force definition is applied consistently across the full study period, we assume that the analyses on time trends in WLE are not affected.

#### 4.3.3 Educational inequalities in WLE

The data contain information on the highest school-leaving qualification, which allowed us to calculate WLE by two educational groups. While using three or more educational groups would allow for a deeper insight in inequalities in WLE, a more detailed classification could not be used due to data restrictions. The information on education is usually available for those who ever had an occupation within the study period, but it is lacking for those who were not (e.g. long-term unemployed and long-term non-labor force). Therefore, the educational information on working spouses or parents have been transferred to non-working family insured individuals with missing information on educational attainment. Beyond theoretical consideration, previous studies have shown that other strongly education-dependent characteristics, such as health status and health inequalities, can be well depicted based on transferred educational information (Muschik et al., 2015; Safieddine et al., 2020). Although the assignment may not be correct in every case, the above mentioned theoretical considerations and previous research (Muschik et al., 2015; Safieddine et al., 2020) suggest that this methodological approach can be used to estimate educational inequalities in the WLE. The robustness of our results is supported by the fact that the educational inequalities found in our study are overall consistent with the findings from previous research (Dudel et al., 2021).

Although the analyses on educational inequalities are limited to the period with the lowest missing proportion (Table 1; Online Resource 1, Table A5), there is some selectivity, as persons with missing educational information had to be excluded from the analyses. The exclusion of these persons led to a higher WLE in the two education groups compared to the whole insurance population (Fig. 4 compared to Fig. 3). This is probably due to the fact that persons with very low education, who often have interrupted employment histories, were disproportionately often excluded from the analyses due to missing information on education. Additional analyses are supporting this assumption, since WLE of individuals without educational information is much lower than in the total insurance population (Online Resource 1, Fig. A6). Furthermore, the data do not allow us to distinguish between persons without a school-leaving certificate and those with missing information, which means that individuals without a school-leaving certificate had also to be excluded from the analyses. This suggests that WLE among the low-educated group tends to be over- and educational inequalities in the WLE tend to be underestimated. However, by using strategies for assigning information on education, e.g. those of the parents or spouses, the effect of excluded individuals with missing information on education could be reduced (Online Resource 1, Fig. A7). Since the proportion of missing values is higher among women and low-educated individuals, the effect of assigning educational information on WLE is strongest for women and low-educated individuals (Online Resource 1, Fig. A8).

For this study, the health insurance data covering 13 years were used, which reduced the share of individuals with missing information on education since it is usually available for individuals who were employed at some point during the observation period. Nonetheless, it must be noted that WLE is likely to be somewhat over- and educational inequalities in WLE to be somewhat under-estimated using the full dataset of 13 years of observation as individuals without school-leaving qualification and low levels of education are likely to be excluded more often due to missing information on education. This issue is the larger, the shorter the period for which the data are available. Thus, the advantages of health insurance data must be weighed against these disadvantages especially if the data cover short observation periods.

### 4.4 Future perspectives and practical implications

In recent decades, great efforts have been made to reduce the impact of population ageing on social security systems and economy. These efforts were most apparent at older ages by current increases in the statutory retirement age. In Germany, the discussion about a further increase in the retirement age is mainly driven by two developments. First, as in other countries, rising life expectancy is leading to an increasing share of life spent outside the labor force. Second, the strong baby boomer generation in Germany is expected to reduce the number of employed individuals in the coming years and exacerbate the shortage of skilled workers (Geyer & Eberhard, 2020). Increasing WLE is considered an essential measure to mitigate the impact of these developments on the economy and society. Given the population ageing and increasing labor shortage, WLE defined as years spent in the labor force provides insight into the potential years in active employment. Not all years in the labor force are spent in active employment. However, similar to the development of life years in the labor force, increases were found in years spent in paid work (Dudel et al., 2021), indicating that a meaningful proportion of the increase in years in the labor force is already spent in active employment. However, in order to counteract the consequences of further population ageing, it is vital to promote further adaptations of the labor market to the needs of older workers.

In addition, the question arises as to whether the health of the ageing populations allows for a (further) extension of working life. This is especially true for countries where labor force participation in old age is already high, such as in Germany. So far, however, research about how healthy life years and healthy working life years develop and influence each other in ageing populations, and whether time trends differ between socio-economic groups, is still insufficient.

From a social policy perspective, these questions are highly relevant, which underlines the importance of exploiting the potentials of existing data sources. Health insurance data represent a still little-used but valuable data source that can expand the previous research which is so far usually based on survey data. This applies especially to the potentials related to the combination of labor force participation with people's health status to study Health Expectancies and Healthy Working Life Expectancy (HWLE), e.g. with regard to specific diseases that are often associated with severe limitations or early retirement. This would be essential knowledge for assessing the possibilities and limits of a further extension of working lives (Boissonneault & Rios, 2021) what could not be investigated with other data sources.

## 5 Conclusion

The study shows that WLE has increased for both genders, suggesting that the policy measures taken to extend working lives are effective. Although the gender gap in WLE has narrowed over time, men still spend considerably more years in the labor force than women. The potential for increasing WLE is particularly high among women with low education. Measures specifically aimed at increasing the employment rate of women with lower education may therefore be particularly promising in further increasing WLE at population level. Moreover, the study suggests that German health insurance data are an appropriate source to calculate educational inequalities in WLE as long as the data are available for a longer time period and the limitations are taken into account. Further research should build on the strengths of the data and investigate whether the observed increase in WLE is also associated with an increase in years in labor free of diseases that are known to be associated with reduced working ability and early exits from the labor market.

## Funding

JE's and JT's work was supported by the AOK Niedersachsen (Statutory Local Health Insurance of Lower Saxony) [grant number: not applicable] as part of an ongoing project on morbidity compression. JE's and FT's work was supported by the German Research Foundation (DFG) [grant number: TE 1395/1-1]. The funders had no role in study design, data collection and analysis, decision to publish, or preparation of the manuscript.

## Ethical statement

Our study is based on claims data, i.e., on routinely collected data of a statutory health insurance provider. We confirm that all data are fully anonymized before we accessed them. The use of this sort of data for scientific purposes is regulated by federal law. The data protection officer of the Statutory Local Health Insurance of Lower Saxony (AOK Niedersachsen) has approved its use.

## CRediT authorship contribution statement

**J. Tetzlaff:** Conceptualization, Methodology, Formal analysis, Data curation, Writing – original draft. **M. Luy:** Writing – review & editing, discussing results and implications. **J. Epping:** Writing – review & editing, discussing results and implications. **S. Geyer:** Supervision, Writing – review & editing, discussing results. **J. Beller:** Writing – review & editing, discussing results. **J.T. Stahmeyer:** Writing – review & editing, discussing results. **S. Sperlich:** Writing – review & editing, discussing results. **F. Tetzlaff:** Methodology, Formal analysis, Data curation, Writing – original draft, Visualization.

## Declaration of competing interest

None.

## Data Availability

The authors do not have permission to share data.
