# Supplementary material for: Estimating trends in working life expectancy based on health insurance data from Germany – Challenges and advantages
Source: SSM Popul Health. 2022 Aug 28;19:101215. doi: 10.1016/j.ssmph.2022.101215 (PMC9450162; doi:10.1016/j.ssmph.2022.101215)
Supplement: Multimedia component 1 [file mmc1.pdf]

Online Resource 1

Estimating Trends in Working Life Expectancy based on Health Insurance Data from Germany – Challenges and Advantages

Tetzlaff J[1)*], Marc Luy[2)3)], Epping J[1)], Geyer S[1)], Beller J[1)], Stahmeyer JT[4)], Sperlich S[1)], Tetzlaff F[1)5)]

[1)] Medical Sociology Unit, Hannover Medical School, Carl-Neuberg-Str.1, Hannover, Germany

[2)] Vienna Institute of Demography, Austrian Academy of Sciences, Vienna, Austria

[3)] Wittgenstein Centre for Demography and Global Human Capital (IIASA, OeAW, University of Vienna), Vienna, Austria

[4)] AOK Niedersachsen- Statutory Health Insurance of Lower Saxony, Hannover, Germany

[5)] Division of Social Determinants of Health, Robert Koch-Institute, Berlin, Germany

*Corresponding author

E-mail: tetzlaff.juliane@mh-hannover.de

Additional remarks 1:
Health insurance fee are equally paid by the employer and the employed person in case of regular employment. The same applies to retired individuals for whom fees are shared between the Statutory Pension Fund and the retired person. For unemployed individuals, health insurance fees are covered by the Federal Employment Agency or by the municipality in case of long-term unemployment.

Additional remarks 2:

Employment histories are well documented in statutory health insurance data due to legal regulations of the German social security system. Employers, the Statutory Pension Fund and the Federal Employment Agency are legally obliged to report on socio-economic characteristics (such as income) and episodes the insured individuals spent in (un-)employment and in retirement to the statutory health insurance provider. Thus, transitions from one employment status to another are well documented in the health insurance data and can be assigned to a specific dates. There are different types of episodes: Compulsorily insured employees, including self-employed insured employees were combined to the group of “employed”. Compulsorily insured employees have a paid employment with salaries subject to the social security contributions. All incomes from salaries above a certain level are subject to the social security contributions (in 2022, 450€ per month). This level is unlikely to be reached without working several hours a week. In the health insurance data, the employment status does not change for persons who are temporarily absent from work, e.g. due to parental leave, illness and further training, which is in line with the ILO definition. Unemployed persons who receive compensations from the mandatory unemployment insurance or social benefits due to long-term unemployment can also be identified in the data and were classified as labour force. In contrast to many survey studies, it cannot be directly deduced from the data whether an individual is actually actively looking for a new job or is available to the labour market in the short term. However, especially long-term federal unemployment benefits are only paid if efforts to return to the labour market are discernible (Book VI of the Social Code II (§2 SGB II. https://www.gesetze-im-internet.de/sgb_2/__2.html). Furthermore, the data contain information on pension payments, for example due to old age or disability. If there was no parallel episode of employment, these episodes were assigned to the non-labour force (inactive). In addition, the data include information on the insurance episodes "family insured" and "in training or student". In the German health insurance system, married partners of working age without own income can be co-insured with their working spouses at no additional fees (family insured). Similarly, young adults below age 23 without own income can be co-insured with a working parent. These episodes of family insurance were also counted to the inactive episodes. The episodes “in training or student” indicate time periods in training for individuals above the age of 23. These episodes were assigned to non-labour force.

Table A1 Working Life Expectancy at age 18, 50 and 60 by period

		Men		Women	
Age	Period	Working Life Expectancy	95%-CI	Working Life Expectancy	95%-CI
18	2006-2008	35.79	35.70-35.86	27.47	27.39-27.56
	2011-2013	36.91	36.84-36.97	30.27	30.18-30.35
	2016-2018	38.33	38.28-38.39	34.02	33.95-34.08
50	2006-2008	10.25	10.20-10.29	7.80	7.76-7.85
	2011-2013	11.01	10.97-11.05	9.14	9.09-9.18
	2016-2018	11.70	11.66-11.74	10.46	10.42-10.50
60	2006-2008	2.80	2.78-2.83	1.96	1.93-1.99
	2011-2013	3.38	3.35-3.34	2.71	2.68-2.73
	2016-2018	3.73	3.71-3.74	3.30	3.28-3.32

Data source: AOK Lower Saxony health insurance data

Figure A2 Working Life Expectancy across age by sex and period

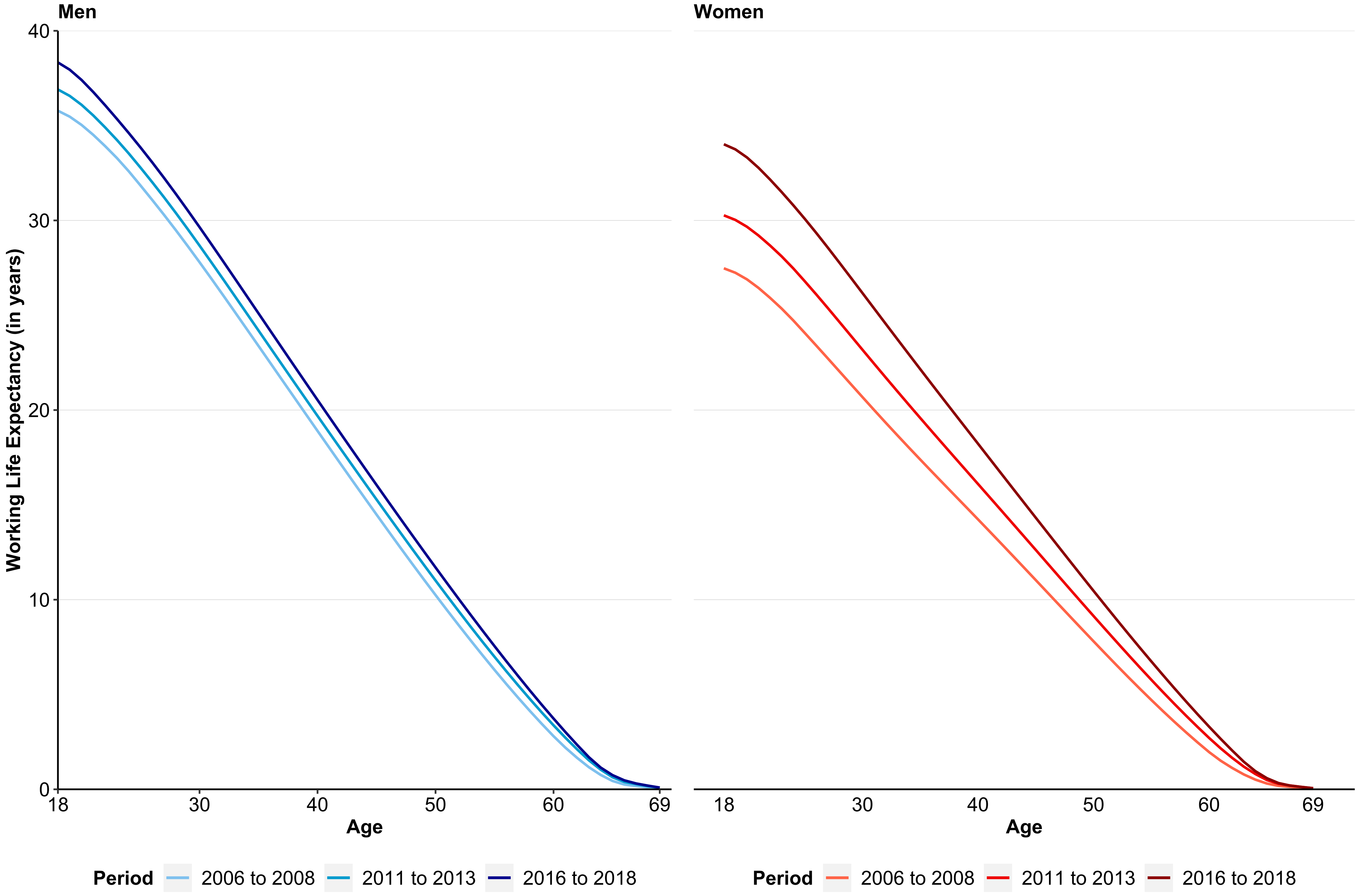

Note: Database contains all individuals insured with the AOK Lower Saxony in the respective period aged 18 to 69 years

Figure A3: Working Life Expectancy across age in 2011-2013 by sex and education

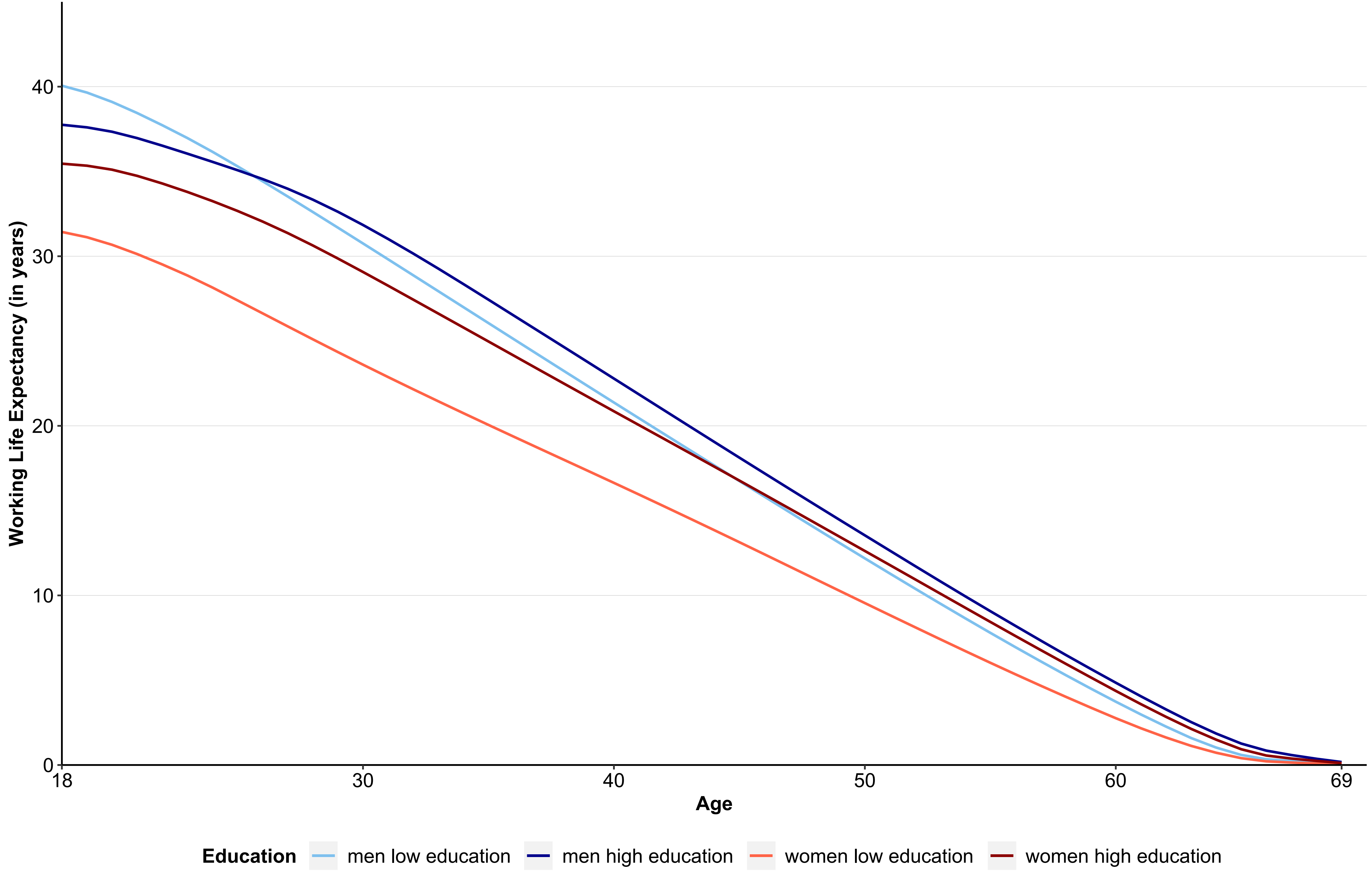

Note: Database contains individuals insured with the AOK Lower Saxony in 2011-2013 aged 18 to 69 years.
Individuals with missing information on education were excluded

Table A4 Working Life Expectancy at age 18 and 50 by education

		Men		Women	
Age	Education	Working Life Expectancy	95%-CI	Working Life Expectancy	95%-CI
18	high	37.75	37.52-38.00	35.46	35.18-35.75
	low	40.06	39.99-40.13	31.43	31.34-31.53
50	high	13.54	13.33-13.75	12.62	12.37-12.87
	low	12.18	12.13-12.23	9.55	9.49-9.60

Data source: AOK Lower Saxony health insurance data

Table A5 Number of individuals and person-years by period and sex without and after assigning educational information to individuals with missing values on education[1)]

Education	Period	Original data				After assigning education to missing values			
		Men		Women		Men		Women	
		Individuals	Person-years	Individuals	Person-years	Individuals	Person-years	Individuals	Person-years
low	2006-2008	576,005	1,147,837	450,548	863,286	607,889	1,198,552	574,096	1,123,236
		55%	54%	43%	41%	58%	57%	55%	53%
	2011-2013	693,226	1,434,192	529,587	1,053,981	732,227	1,501,371	671,877	1,359,508
		58%	59%	47%	45%	61%	62%	59%	59%
	2016-2018	741,972	1,543,208	569,414	1,154,656	801,683	1,641,891	747,470	1,507,678
		53%	56%	43%	44%	57%	60%	56%	58%
high	2006-2008	86,640	149,121	96,526	170,467	90,447	155,633	104,782	186,417
		8%	7%	9%	8%	9%	7%	10%	9%
	2011-2013	129,498	226,355	146,681	260,592	135,036	235,775	158,468	283,417
		11%	9%	13%	11%	11%	10%	14%	12%
	2016-2018	163,454	287,702	189,085	335,970	173,902	304,406	210,049	372,554
		12%	11%	14%	13%	12%	11%	16%	14%
missing	2006-2008	380,748	817,039	490,278	1,091,949	345,057	759,812	358,474	816,050
		36%	39%	47%	51%	33%	36%	35%	38%
	2011-2013	370,337	759,583	461,473	1,007,176	325,798	682,985	307,396	678,825
		31%	31%	41%	43%	27%	28%	27%	29%
	2016-2018	501,132	906,294	565,290	1,107,784	430,973	790,908	366,270	718,178
		36%	33%	43%	43%	31%	29%	28%	28%

Note: [1)]The procedure for assigning educational information to individuals with missing values is described in detail in the methods section of manuscript; Data Source AOK Lower Saxony Health insurance data

Figure A6 Working Life Expectancy of Individuals with missing information on education across age by sex and period

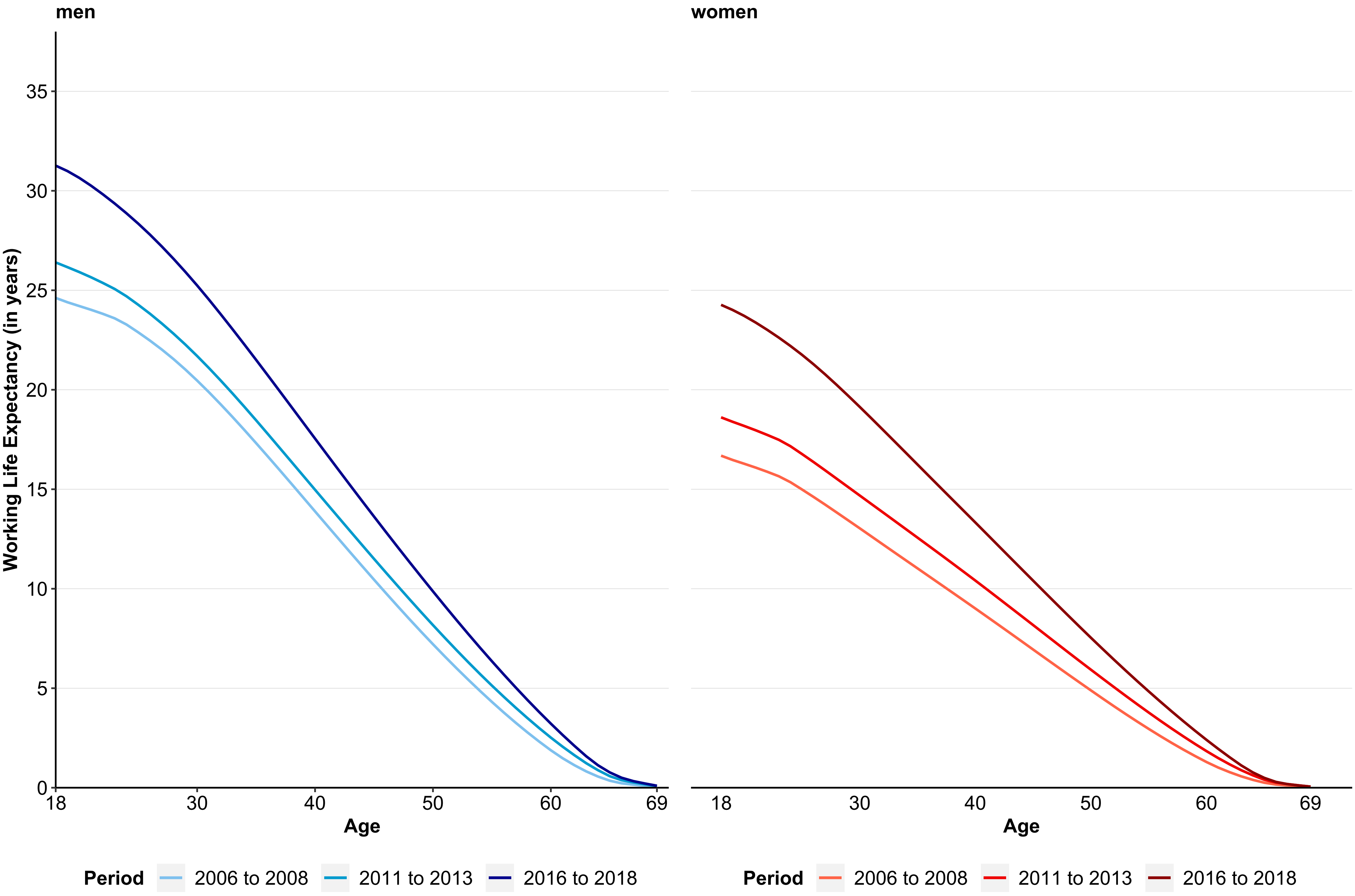

Note: data source AOK Lower Saxony health insurance data

Figure A7 Educational inequalities in Working Life Expectancy without and after assigning educational information to individuals with missing information on education by sex*

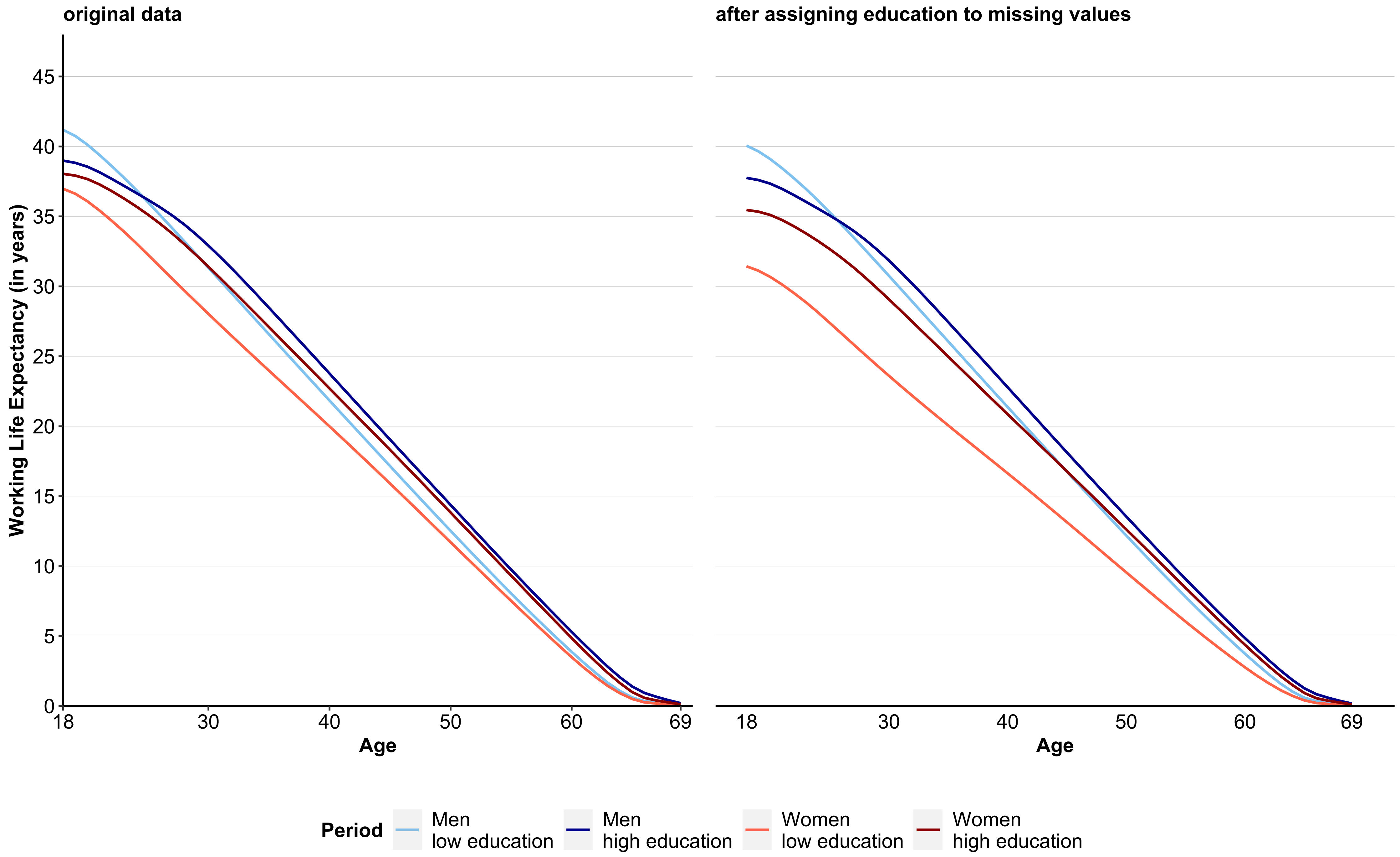

Note: *The procedure for assigning educational information to individuals with missing values is described in detail in the method section of the manuscript
data source AOK Lower Saxony health insurence data

Figure A8 Working Life Expectancy without and after assigning educational information to individuals with missing information on education* across age by educational group and sex

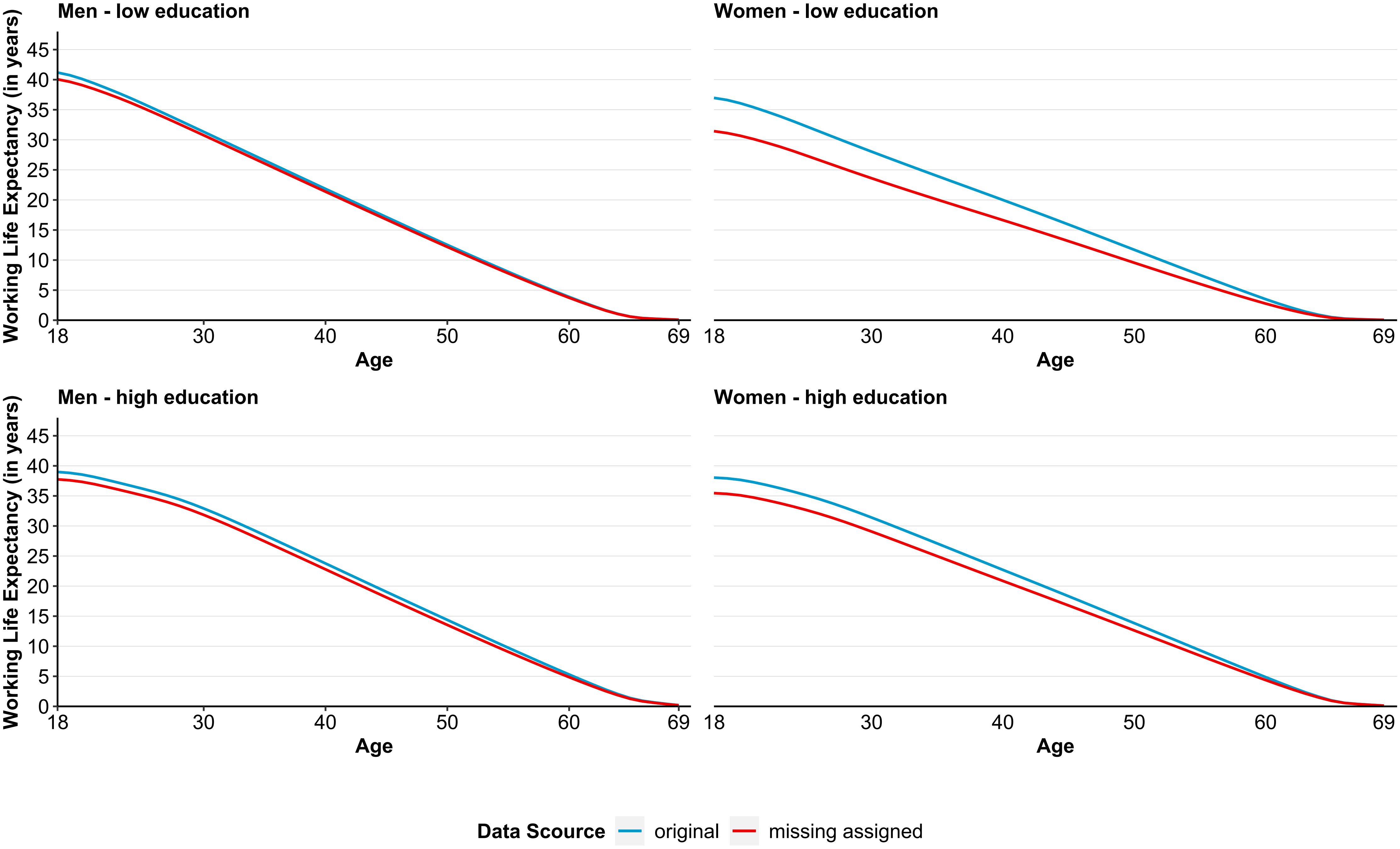

Note: *The procedure for assigning educational information to individuals with missing values is described in detail in the method section of the manuscript
data source AOK Lower Saxony health insurence data
